# Real-Time Measurement Technology of Bearing Housing Clearance in a Rolling Mill

**DOI:** 10.3390/s25061887

**Published:** 2025-03-18

**Authors:** Jiankang Xing, Yan Peng, Xiangyang Zhao, Xinxiang Hou

**Affiliations:** National Engineering Research Center for Equipment and Technology of Cold Strip Rolling, Yanshan University, Qinhuangdao 066004, China; xjk@ysu.edu.cn (J.X.); zhxy.ysu@outlook.com (X.Z.); houxinxiang_ysu@163.com (X.H.)

**Keywords:** strip mill, measurement technology, assembly clearance, control strategy, bearing housing

## Abstract

The assembly clearance between the bearing housing and rolling mill stand affects the roll change and rolling stability. In order to improve the accuracy and real-time measurement of the bearing housing clearance of the rolling mill, four kinds of measuring methods were designed, namely the laser ranging method, external force measuring method, internal force measuring method, and eddy current ranging method, and the characteristics of the four measuring methods were introduced, respectively. The real-time measuring experiment of bearing housing clearance was carried out in a 100 mm two-high mill in laboratory and a 1580 mm four-high hot tandem mill in the Qian’an Iron and Steel Company. The results show that clearance measurement technology is helpful to improve the accuracy of real-time measurements and can provide guidance for the clearance control work. Finally, based on the real-time measurement technology of bearing housing clearance, the control strategy of bearing housing clearance was developed. This technology is of great significance to realize the fine management of rolling mill clearance and to improve the intelligence level of rolling mill systems.

## 1. Introduction

Strip steel is widely used in various types of steel for application in many sectors. The development level of the iron and steel industry reflects a country’s scientific and technological level and economic strength to a great extent, and the production level of strip steel usually reflects the development level of a country’s iron and steel industry [1,2,3,4].

The reasonable design of the assembly clearance between the bearing housing and the rolling mill stand is a prerequisite for the rolling mill to be able to function normally [5,6,7,8,9]. For strip mills, if the clearance is too small, the roll cannot be installed into the rolling mill stand or cannot be removed smoothly after installation, thus affecting the normal roll change. If the clearance is too large, it will affect the horizontal stiffness of the rolling mill, which will cause the rolling mill vibration to intensify. Moreover, it will cause obvious thickness differences in the strip or bright and dark stripes on the surface of the strip, which seriously affects the product quality. Ultimately, it will have a negative impact on equipment maintenance, rolling stability, and product quality [10,11,12,13,14].

The working environment of the rolling mill is very harsh. In the production process, the bearing housing liner plates, the rolling mill stand liner plates, the mounting surface of the bearing housing, and the rolling mill stand will have different degrees of wear, which makes the clearance between the bearing housing and the rolling mill stand gradually larger, further aggravating the wear of the liner plates [15,16]. The uneven wear of the liner plates of the bearing housing of the mill will also cause the phenomenon of roll crossing. This will aggravate the vibration of the mill and affect the shape quality of the product [17,18]. When the liner plates are seriously worn, causing the clearance to exceed the standard allowable range, they must be repaired in time. Generally, this problem will be solved using padding. Therefore, the clearance measurement between the bearing housing and the rolling mill stand is very important for equipment maintenance [19,20,21,22,23].

The traditional measurement method of the clearance is to measure the width of the bearing housing with an outer diameter micrometer to obtain the width between the two liner plates of the bearing housing, and then to measure the distance between the two liner plates of the rolling mill stand with an inner diameter micrometer to obtain the distance between the two liner plates of the rolling mill stand (also known as the opening size). The opening size of the rolling mill stand minus the width of the bearing housing is the clearance value between the bearing housing and the rolling mill stand. However, this method is affected by human factors and has a large measurement error. At present, a 3D laser tracker is generally used in industrial fields to measure the opening size of the rolling mill stand, the width of the bearing housing, and the flatness and symmetry of liner plates, and then to obtain clearance information on the bearing housing and the rolling mill stand. This method has high measurement accuracy, so it is widely used in steel mills.

Yang, J. [24] introduced the measurement principle of the laser tracker and made a comparative analysis of the traditional measurement method and the laser tracker measurement method of the rolling mill clearance, pointing out that the laser tracker has higher accuracy. Song, G. [25] used the laser tracker to measure the spatial position of the rolls under actual working conditions, established a geometric correlation model between liner wear and roll system cross angle according to the spatial geometric state of the liner, and considered the influence of the amount of rolling mill assembly clearance, which gave the range of roll system cross angles under actual working conditions. Zhang, Z. [26] used a laser tracker to measure the spatial position of the liner surface of the bearing housing and obtained the inclination value caused by liner wear and the cross value of the upper and lower work rollers. Based on 3D laser measurement technology, Lu, Q. [27] studied the influence of the spatial crossing of the roll system on the stability of the rolling mill and pointed out that the measurement of the clearance of the rolling mill has an important guiding role in the adjustment of the liner. Shen, S. [28] introduced the measurement method of using a Leica AT500 laser tracker and analyzed the influencing factors of measurement accuracy. He pointed out that the application of a laser tracker is of great significance to guarantee the accuracy of equipment. Gao, Y. [29] introduced the measuring steps of a laser tracker and analyzed the measuring data. The results showed that there was a roll crossing phenomenon in the rolling mill. Xie, R. [30] made a comprehensive measurement of the four-high mill stand with a laser tracker and repaired the rolling mill stand liner according to the measurement results, so that the offset of the rolling mill stand center line was controlled within a small range and the problem of excessive axial force of the roller was solved. Wei, X. [31] used a laser tracker to measure the stand of a 1550 mm four-high mill and pointed out that the measurement data can provide guidance on the adjustment of the liner.

The traditional measurement method and the laser tracker measurement method have the following drawbacks. First of all, the measurement results are the clearance information of the rolling mill in the offline state, and the equipment state information in the rolling process cannot be obtained. Secondly, the width of the bearing housing and the opening size of the rolling mill stand are measured periodically, which cannot guarantee real-time clearance control, and these measurement require production to be halted, which affects the economic state of the enterprise. Thirdly, because the clearance data cannot be obtained in real time, the establishment of the clearance control standard is mainly based on experience. In order to avoid situations where the roll cannot be installed into the rolling mill stand or cannot be removed smoothly when the roll needs to be changed, the clearance control standard is generally too large, which will lead to abnormal vibration of the rolling mill.

In summary, at present, all methods for measuring this clearance require equipment shutdown. The real-time measurement of the clearance information cannot be guaranteed, and the shutdown measurement directly affects the economic state of enterprises. Real-time measurement technology of the bearing housing clearance can obtain the clearance between the bearing housing and the rolling mill stand while the equipment is online, which has an important guiding role in the formulation of clearance control strategy and is of great significance for reducing rolling mill vibration, reducing roll wear, and improving product quality.

## 2. Real-Time Measurement Method of Bearing Housing Clearance in Rolling Mills

Aiming at the problem that the clearance between the bearing housing and the rolling mill stand cannot be measured while the equipment is online, four methods to measure the clearance in real time were designed. The laser ranging method can be used in laboratory rolling mills, but it is not suitable for industrial rolling mills. In order to realize real-time clearance measurements in industrial rolling mills, three methods have been proposed, namely the external force measuring method, internal force measuring method, and eddy current ranging method. The external force measuring method and the internal force measuring method are in the theoretical research stage. We only demonstrated the theoretical feasibility of these two methods and their respective characteristics, and the specific application effect still needs to be tested in practice. On the basis of the study of these two methods, the eddy current ranging method was proposed, and industrial experiments have verified that the method is suitable for application in industrial rolling mills for real-time clearance measurement.

### 2.1. Laser Ranging Method

The laser displacement sensor is installed on the hydraulic cylinder for the bending rolls of the rolling mill. However, if the application is in a rolling mill without a hydraulic cylinder for the bending rolls, the laser displacement sensor is installed on the rolling mill stand. Because the same bearing housing has the same displacement value measured on the inlet side and the outlet side of the mill, it can be installed on only one side, such as the outlet side of the mill. The installation of the sensor should ensure its stiffness and non-loosening, and the measurement direction of the sensor must be in the horizontal direction. The measuring range of the sensor is greater than 2 mm, and the measuring accuracy is less than 1 μm. We select or install a detection area on the bearing housing to ensure that the light spot of the sensor is in the detection area during the vertical or axial movement of the roll. The surface of the detection area should be kept clean, and the surface roughness should not exceed Ra0.8 μm. We adjust the position of the sensor or the detection area so that the detection area is near the middle of the sensor range.

**Figure 1:**
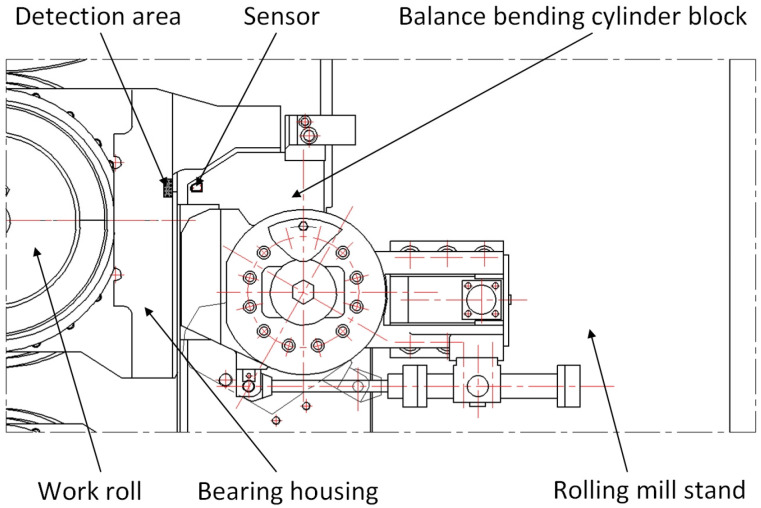
Schematic diagram of laser ranging method.

Before measurement, on the standard displacement test bench, the linear motor or the stepper motor drives the ball screw to provide the standard displacement. The sensitivity coefficient of the sensor is calibrated by the output signals corresponding to different standard displacements. The zero point of the sensor is calibrated by pressing the bearing housing liner against the rolling mill stand liner. During the rolling process, the sensor measures the distance from the sensor to the surface of the detection area in real time. The measured distance can reflect the horizontal displacement of the bearing housing, which can reflect the real-time clearance between the bearing housing and the rolling mill stand, as shown in Figure 1.

This method is simple to implement and can measure the clearance between the bearing housing and the rolling mill stand in real time. However, the measurement accuracy of the sensor will be affected by the cooling water in the industrial field. Additionally, large amounts of cooling water can damage the sensors. Therefore, this method is more suitable for the laboratory rolling mill.

### 2.2. External Force Measuring Method

**Figure 2:**
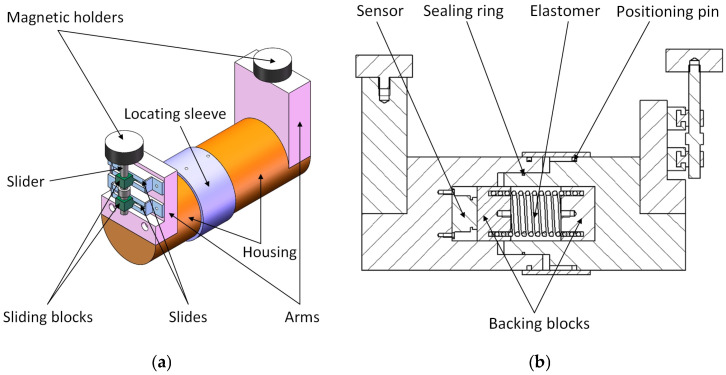
External force measuring element. (**a**) The main structure of the measuring element; (**b**) section view of the main structure of the measuring element.

A measuring element for real-time clearance measurement was designed, including a magnetic holder, housing, positioning pin, slider, sensor, elastomer, sealing ring, etc., as shown in Figure 2.

**Figure 3:**
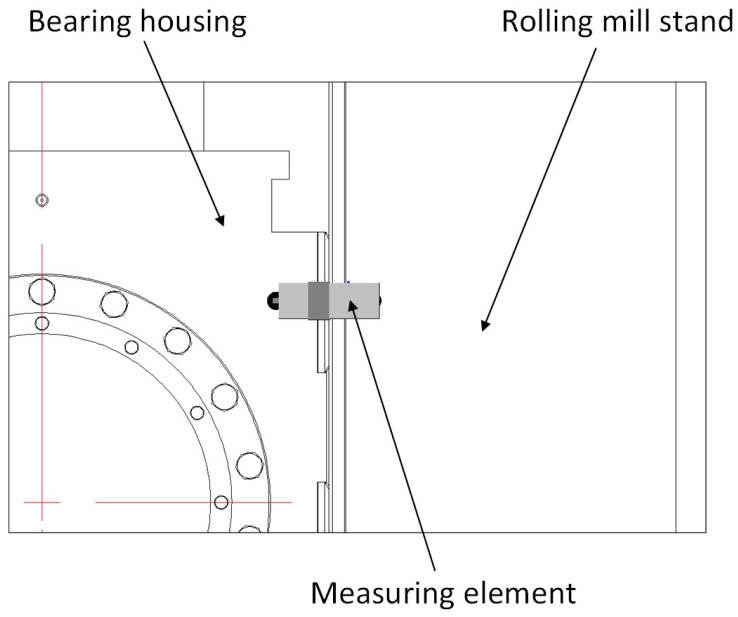
Schematic diagram of the measuring element layout.

Before measurement, the measuring element should be calibrated for its sensitivity coefficient and zero point in a similar way to the calibration of the laser ranging method. During measurement, the two magnetic holders are affixed to the bearing housing and the rolling mill stand (or the balance bending roller cylinder block), as shown in Figure 3. The fixed position should be polished clean, and the axis of the measuring element should be arranged in the horizontal direction. As the rolling mill operates, the horizontal displacement change of bearing housing is transmitted to the elastomer through the magnetic seat and housing. The elastomer converts the change in displacement into a change in force, and the sensor measures this change in force and outputs the corresponding signal. Ultimately, the change in force measured by the sensor reflects the displacement change of the bearing housing, which can reflect the real-time clearance between the bearing housing and the rolling mill stand.

This method can avoid cooling water damage to the sensor, and cooling water or water vapor does not affect its measurement accuracy, so it can work stably for a long time under harsh conditions in industrial fields. However, the measuring element must be removed and reinstalled every time the roller is changed, and frequent roller changes in the rolling mill cause inconvenience to the application of the measuring element in the industrial field. This method can theoretically be used for the real-time measurement of bearing housing clearance in rolling mills. However, the specific application effect needs to be tested in practice.

### 2.3. Internal Force Measuring Method

**Figure 4:**
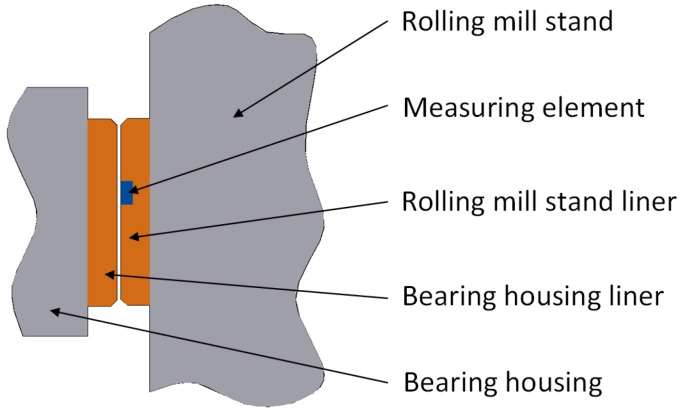
Installation diagram of the measuring element.

Another measuring element for real-time clearance measurement was designed. During the measurement process, the measuring element is installed in the rolling mill stand liner plate, as shown in Figure 4. The installation position of the measuring element should take into account the stroke of the mill press-down and the axial movement of the roll to avoid measurement errors caused by the sensor detecting the position of the fixing bolt holes and oil grooves on the corresponding bearing housing liner during the measurement process.

**Figure 5:**
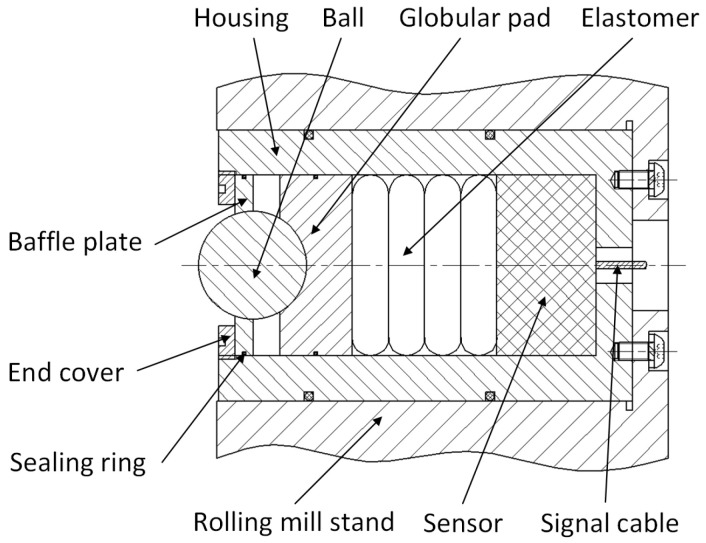
Internal force measuring element.

The measuring element includes a housing, ball, sealing ring, globular pad, elastomer, sensor, etc., as shown in Figure 5. Before measurement, the measuring element should be calibrated for the sensitivity coefficient and zero point in a similar way as described above. When the roller is removed, the ball in the measuring element will be ejected by the action of the elastomer, and the baffle will act as a limiter. When the roller is installed in the rolling mill stand, the ball will be pressed into the measuring element by the bearing housing liner. During the functioning of rolling mill, the horizontal displacement change in the bearing housing liner will be transmitted to the elastomer through the ball and globular pad. The elastomer converts the displacement change into a force change, and the sensor outputs a real-time force signal. The change in force measured by the sensor can reflect the change in the displacement of the bearing housing and can finally achieve the real-time measurement of the clearance information between the bearing housing and the rolling mill stand.

This method can also theoretically be used for the real-time measurement of bearing housing clearance in a rolling mill and is also unaffected by water and water vapor in the environment. Moreover, this method is not affected by frequent roller changes, so it is more suitable for applications in industrial fields. Nonetheless, the specific application effect also needs to be tested in future practice.

### 2.4. Eddy Current Ranging Method

**Figure 6:**
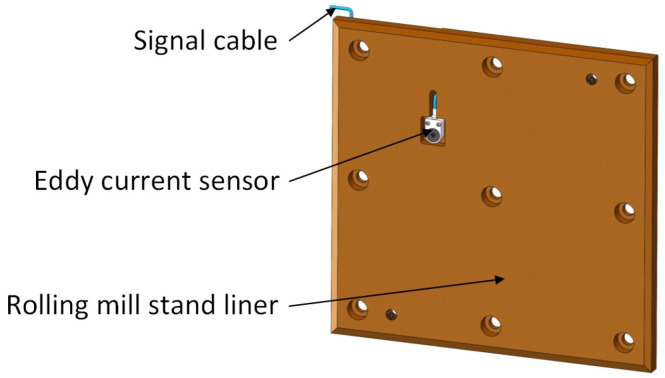
Installation diagram of eddy current sensor.

Based on the research on the external force measuring method and internal force measuring method, an eddy current ranging method was proposed. Similar to the internal force measuring method, it is necessary to replace the measuring element inside the rolling mill stand liner in Figure 4 with an eddy current sensor. A sensor mounting slot is machined on the outside of the rolling mill stand liner, as shown in Figure 6. The size of the mounting slot should be three times larger than the diameter of the sensor probe to prevent the metal part of the side of the mounting slot from affecting the measurement accuracy of the eddy current sensor. The depth of the slot must be 0.2–0.5 mm larger than the thickness of the sensor. Two M2–M4 thread holes must be prepared on the bottom of the slot for installing the sensor. We drill a through hole on the bottom of the mounting slot and machine a cable trough inside the rolling mill stand liner. The cable of the sensor passes through the hole, leads out the liner through the cable trough, and connects to the signal acquisition device. The location of the mounting slot should be selected by taking into account the stroke of the mill press-down and the axial movement of the roll to avoid measurement errors caused by the sensor detecting the position of the fixing bolt holes and oil grooves on the corresponding bearing housing liner during the measurement process.

The sensor should have a thickness of 10–20 mm, a range of 2–4 mm, a measuring accuracy of less than 1 μm and a protection class of IP67. The sensor should be calibrated before installation. The size of the metal plate used for calibration should be ≥100 × 100 mm, the thickness should be ≥10 mm, and the material is the same as the material of the bearing housing liner. During the measurement process, the sensor measures the distance from the sensor to the bearing housing liner. Through calculation, the clearance value between the rolling mill stand liner and the bearing housing liner can be obtained. If the rolling mill is equipped with several sets of roll bearing housings of different materials, it is necessary to mark the roll number according to the liner material, and the measurement of clearance needs to be corrected by using the correction factor for rolls of different materials.

This method can be used for the real-time measurement of the bearing housing clearance in an industrial rolling mill. Furthermore, this method is unaffected by water and frequent roller changes.

## 3. Experiments on the Real-Time Measurement of Bearing Housing Clearance

The above four real-time measurement methods for rolling mill bearing housing clearance can avoid the negative impact on products and equipment caused by excessive clearance without requiring a facility maintenance period or the cessation of production. Because the clearance information can be measured in real time, the rolling mill clearance control standard can be further optimized, which is conducive to the stability of rolling mill production. Moreover, the real-time horizontal displacement information of the bearing housing can reflect the horizontal vibration of the rolling mill, and fault diagnosis can be made according to the amplitude and frequency of the horizontal displacement signal during the production process.

Of the four measurement methods, the laser ranging method is the simplest to implement. Although it cannot be applied in industrial fields, it is a good choice to use in laboratory rolling mills. The eddy current ranging method is the best choice for industrial rolling mills because of its simple structure and its applicability to the harsh working conditions in industrial fields. Therefore, experiments were carried out on a laboratory 100 mm two-high mill using the laser ranging method and on an industrial mill using the eddy current ranging method, respectively.

### 3.1. Laboratory Experiment

**Figure 7:**
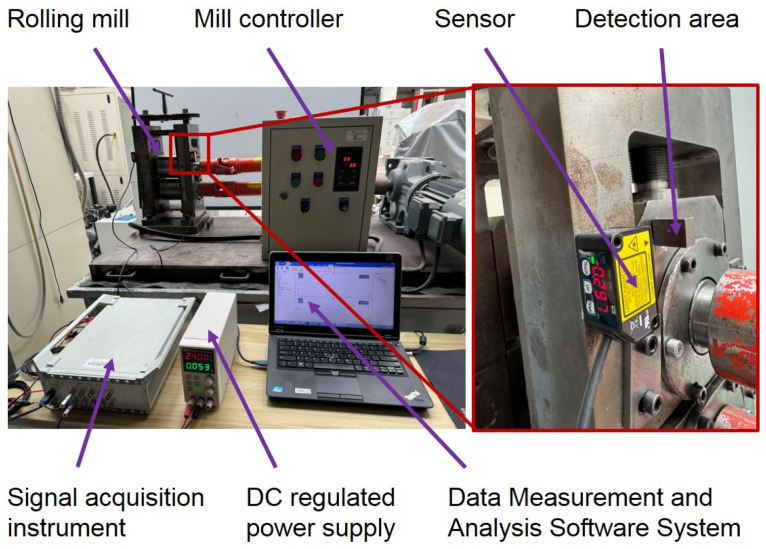
Experiments on the 100 mm rolling mill in the laboratory.

In the laboratory of the 100 mm two-high mill, the measuring experiment of the bearing housing clearance in real time was carried out by using the laser ranging method. The laboratory rolling environment was relatively good, and the experimental rolling mill did not use cooling water, so the measurement accuracy of the laser displacement sensor can be guaranteed. Two laser displacement sensors were mounted on the drive side and the operation side of the rolling mill stand, as shown in Figure 7.

**Figure 8:**
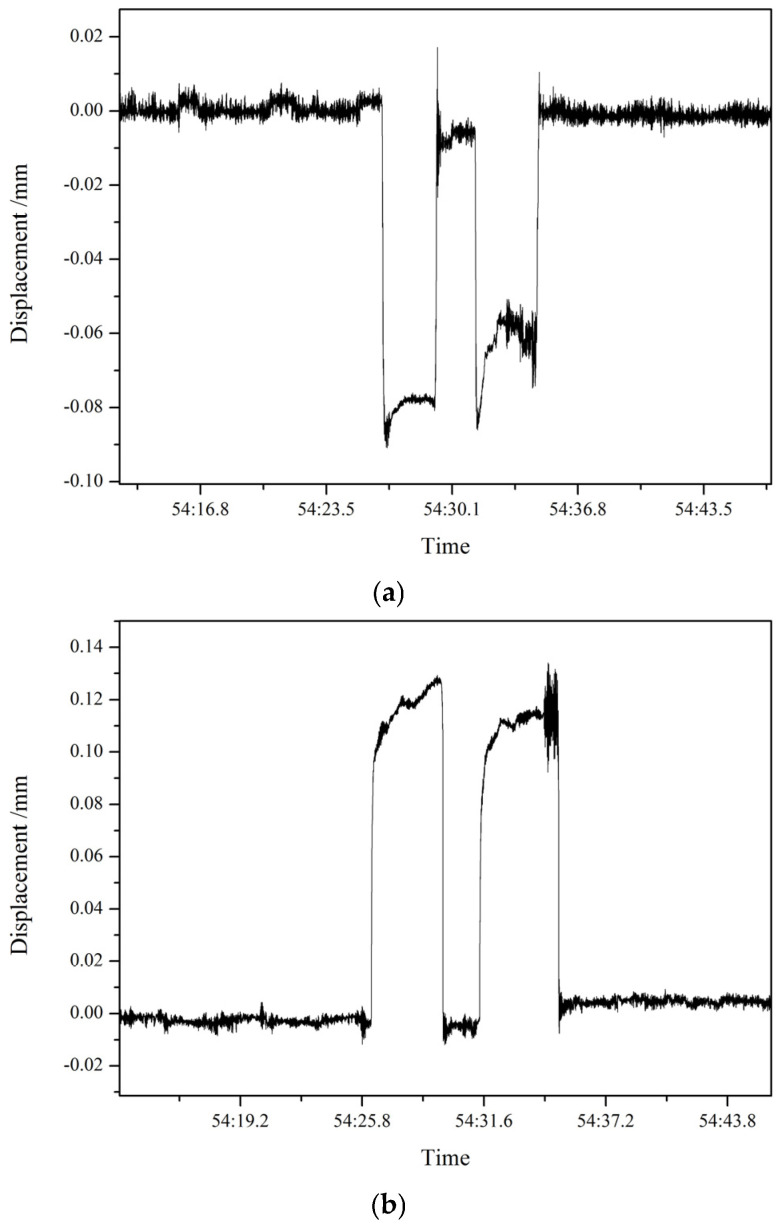
Displacement of the upper work roll bearing housing. (**a**) Displacement of the bearing housing on the drive side of the upper work roll; (**b**) Displacement of the bearing housing on the operation side of the upper work roll.

Rolling experiments were carried out with aluminum plates, and the displacement of the bearing housing on the drive side and the operation side of the upper work roll was monitored in real time during rolling, as shown in Figure 8. It can be seen from the figure that under no-load and load conditions, the bearing housing on the drive side of the upper work roll had a displacement of about 0.09 mm, the bearing housing on the operation side had a displacement of about 0.13 mm, and the displacement directions of the drive side and the operation side of the roll were opposite during the rolling process. As the experimental rolling mill is a two-high mill, the work roll has no lateral constraint caused by the offset between the work roll and the back-up roll, and it is easy to have the opposite displacement direction of the bearing housings on both sides of the roll during rolling. In the four-high mill in the industrial field, the displacement direction of the bearing housings on both sides is generally the same due to the existence of offset. The experiment shows that this measurement method can obtain the clearance between the bearing housing and the rolling mill stand in real time, and that it can track the dynamic change in the position of each bearing housing while the equipment is online, which has important guiding significance for the regulation of the clearance.

**Figure 9:**
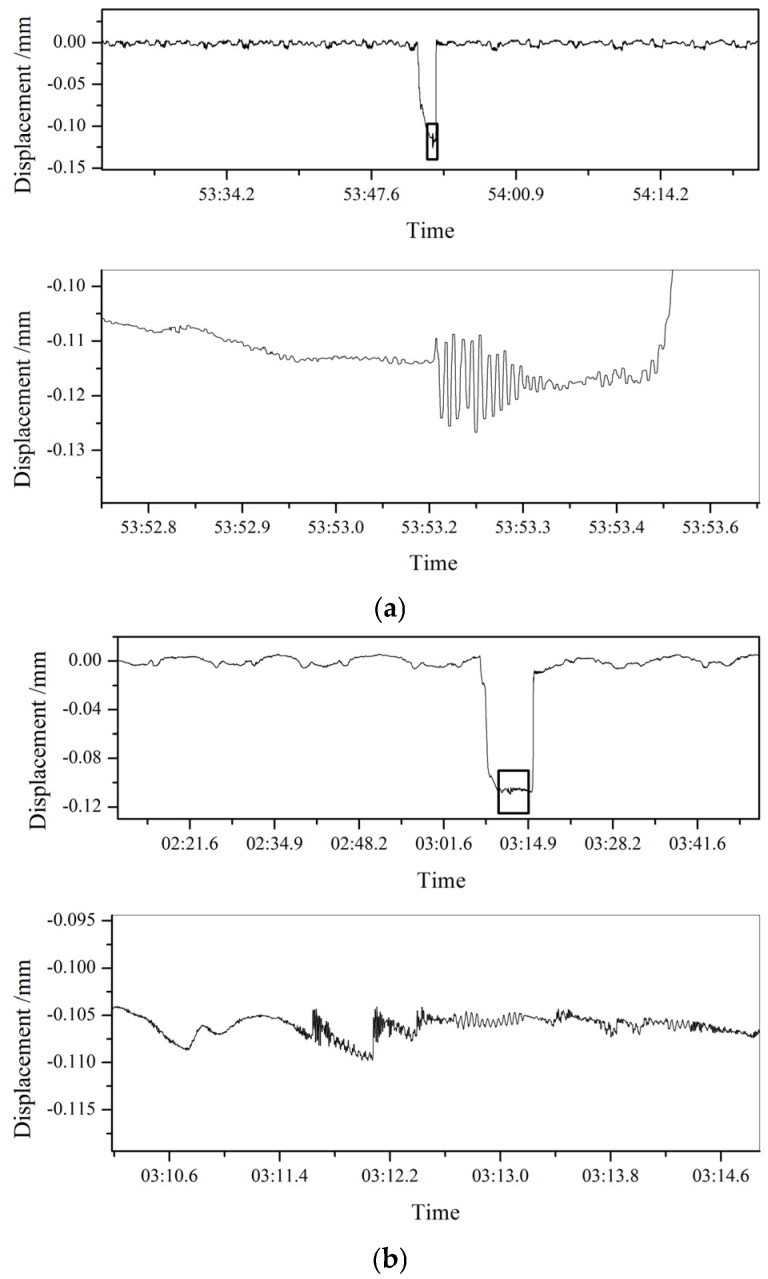
Horizontal vibration amplitude of the bearing housing on the drive side of the upper work roll. (**a**) Rolling speed: 110 mm/s; (**b**) rolling speed: 25 mm/s.

The experiments with different rolling speeds were carried out on the experimental rolling mill. The materials and specifications of the plates used in the experiment were the same and were rolled with the same roll gap. The speed of the first rolling experiment was 110 mm/s and the speed of the second rolling experiment was 25 mm/s. The real-time displacement information of the bearing housing on the drive side of the upper work roll in the rolling process was analyzed, as shown in Figure 9. From the figure, it can be seen that at the higher rolling speed (110 mm/s), the horizontal vibration of the work roll during the rolling process was relatively intense, and the peak-to-peak value of the vibration was about 0.02 mm. However, at a lower rolling speed (25 mm/s), the horizontal vibration of the work roll during the rolling process was relatively stable, and the peak-to-peak value of the vibration was only about 0.006 mm. It can be seen that different rolling processes directly affected the stability of rolling and that higher rolling speeds usually intensified the vibration of the mill.

**Figure 10:**
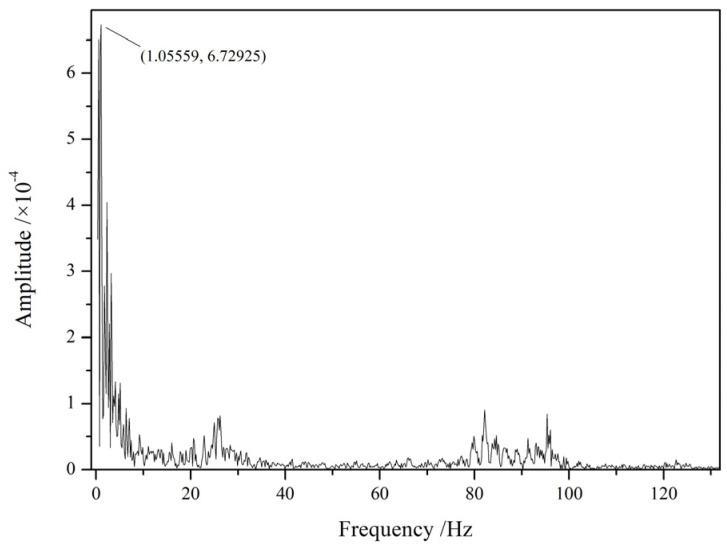
Horizontal vibration spectrum of the bearing housing on the drive side of the upper work roll.

The vibration frequency of the work roll can be obtained by the Fourier transform of the measured signal, as shown in Figure 10. Therefore, this measurement method can not only obtain the clearance of bearing housing in real time but can also diagnose the equipment fault. It can also guide the formulation of rolling technology, so that rolling production can avoid the resonance frequency of rolling mills, which, in turn, has an important significance for reducing mill vibration, reduce roll wear, and improving product quality.

### 3.2. Industrial Experiment

**Figure 11:**
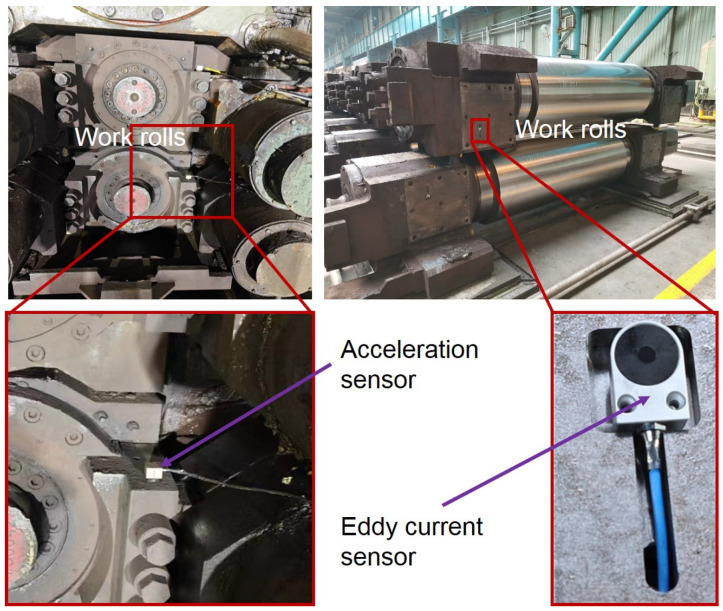
Experiments on a 1580 mm rolling mill in an industrial field.

The real-time measuring experiment of bearing housing clearance was carried out on F3 of a 1580 mm hot tandem mill in Qian’an Iron and Steel Company (Tangshan, China) by using the eddy current ranging method. If the eddy current sensor is mounted on the rolling mill stand liner, it is necessary to stop the rolling mill to remove the liner to process the sensor mounting slot, which will seriously affect the normal production of the rolling mill. Therefore, a sensor was installed on the bearing housing liner. The sensor detects the position of the rolling mill stand liner, and then the clearance information between the bearing housing and the rolling mill stand can be obtained through calculation. All mills are equipped with multiple sets of rolls for replacement when the rolls are badly worn. The replaced rolls go into the grinding workshop for sharpening, after which they are used to prepare for the replacement of the next set of badly worn rolls. Therefore, as long as the sensor mounting slots and cable slots were machined on the offline roll bearing housings, the sensors could be installed. Real-time clearance measurement could be carried out after the rolls equipped with sensors were installed in the mill stand during the roll changes. The installation of sensors in the industrial field is shown in Figure 11.

Therefore, this method does not affect the normal production of the rolling mill and is very convenient for exploratory experiments. However, the roll change process requires sensor communication cable removal and installation. Furthermore, real-time measurement of the bearing housing clearance is only possible when the rolls on which the sensors are mounted are used for production. Therefore, if a long-term real-time measurement of the bearing housing clearance is desired that does not interfere with roll changes, the sensors should be mounted on the mill stand liners.

**Figure 12:**
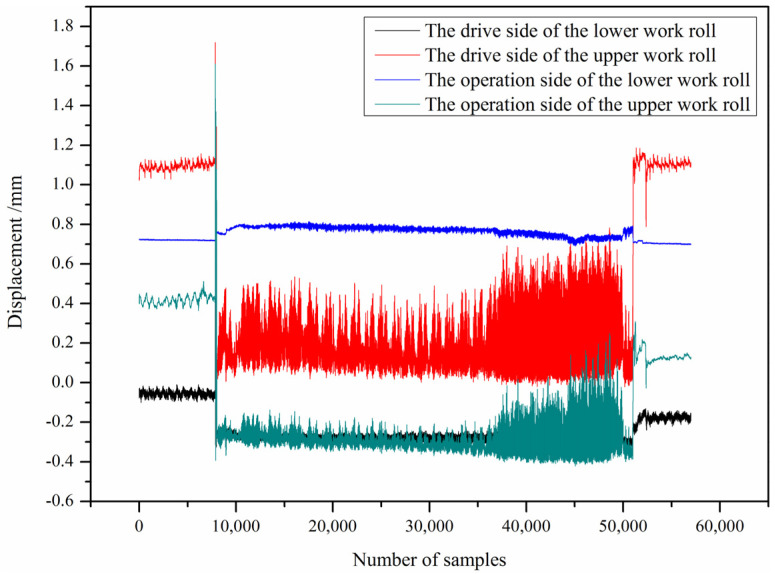
Displacement of the work roll bearing housing.

The industrial experiment monitored the clearance of the bearing housing on the drive side and the operation side of the upper and lower work rolls when rolling a certain steel plate in real time, as shown in Figure 12. From the figure, it can be seen that the displacement of the bearing housing on the drive side and operation side of the lower work roll was small, while the displacement of the bearing housing on the drive side and operation side of the upper work roll was larger. The results show that there w frequent swinging of the upper work roll in the rolling process, and the clearance between the upper work roll bearing housing and the mill stand was large, which needs to be adjusted to improve the stability of the rolling process.

## 4. Control Strategy of Bearing Housing Clearance in Rolling Mills

**Figure 13:**
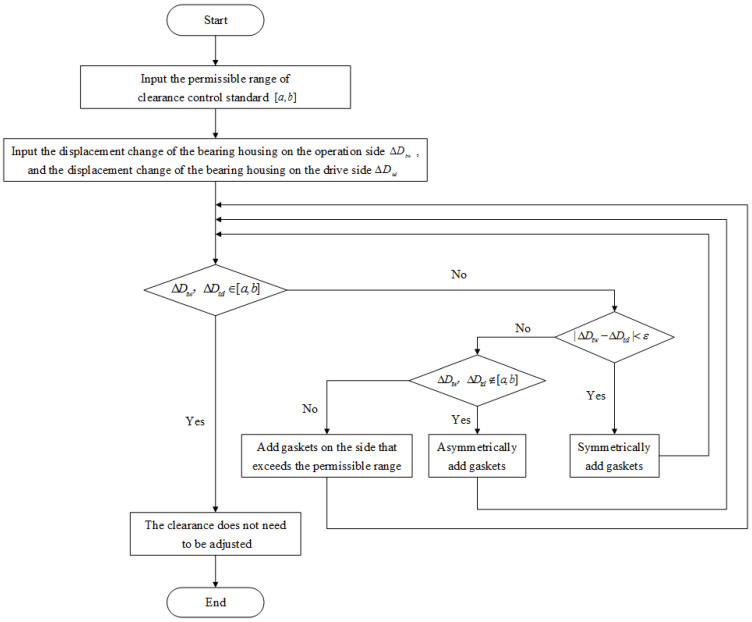
Flow chart of the clearance control strategy.

Because the clearance between the rolling mill bearing housing and the mill stand can be measured in real time by using the abovementioned measurement methods, the clearance control standard can be further reduced on the basis of the original clearance control standard. Generally, the clearance between the rolling mill bearing housing and the mill stand should be controlled within 1 mm. Taking the upper work roll of the mill as an example, the permissible range of clearance control standard after optimization is [a,b], and it is assumed that the spatial position of the lower work roll is fixed. Based on the real-time measured clearance information, the absolute value $\Delta D_{tw}$ of the displacement change in the bearing housing on the operation side of the upper work roll and the absolute value $\Delta D_{td}$ of the displacement change in the bearing housing on the drive side are analyzed between the unloading and the rolling process. Assuming that the minimum thickness of the gasket used in adjusting the clearance is ε, the clearance control strategy between the upper work roll bearing housing and the mill stand is shown in Figure 13.

(1) If both $\Delta D_{tw}$ and $\Delta D_{td}$ are in the permissible range of the clearance control standard, it indicates that the clearance between the upper work roll operation side and drive side bearing housings and the mill stand is in good condition and does not need to be adjusted.

(2) If $\Delta D_{tw}$ and $\Delta D_{td}$ are not both in the permissible range of the clearance control standard, it is necessary to compare $\left|\Delta D_{tw}-\Delta D_{td}\right|$ and ε. If $|\Delta D_{tw}-\Delta D_{td}|<\varepsilon$, it indicates that the clearance between the upper work roll operation side and drive side bearing housings and the mill stand is too large, and it is necessary to symmetrically add gaskets on the operation side and the drive side bearing housings to adjust the clearance to the permissible range of the control standard.

(3) If $\Delta D_{tw}$ and $\Delta D_{td}$ are not both in the permissible range of the clearance control standard, and $|\Delta D_{tw}-\Delta D_{td}|\geq \varepsilon$, it is necessary to continue to determine whether $\Delta D_{tw}$ and $\Delta D_{td}$ are both outside the permissible range [a,b] of the clearance control standard. If $\Delta D_{tw}$ and $\Delta D_{td}$ are not within the range of [a,b], it indicates that the upper and lower work rolls appear to be crossed, and it is necessary to asymmetrically add gaskets on the bearing housing of the operation side and the drive side and to adjust the clearance to the permissible range of the control standard and make the clearance value on both sides equal. Otherwise, it shows that one of $\Delta D_{tw}$ and $\Delta D_{td}$ is in the permissible range of the clearance control standard, and the other side exceeds the permissible range of the clearance control standard, and the upper and lower work rolls appear to be crossed. It is necessary to add gaskets to the bearing housing on the side that exceeds the permissible range of the clearance control standard in order to adjust the clearance to the permissible range of the control standard and to make the clearance values on both sides equal.

The control strategy of the clearance between the bearing housing and the mill stand of the lower work roll is similar to that of the upper work roll. According to the offset design theory of roll layout in four-high plate rolling mill, there is an offset between the work rolls and the back-up rolls, and the work rolls are generally offset to the exit side of the rolling mill. This design allows the work rolls and the backup rolls to be pressed against both sides of the mill stand during the rolling process, without back-and-forth movement and crossing. Therefore, in general, the displacement change direction of the bearing housing on the operation side and the drive side of the upper and lower work rolls are the same, that is, the work rolls are offset to the same side of the mill during the rolling process, usually the exit side of the rolling mill. If the direction is different, it means that the upper and lower work rollers are seriously crossed and the equipment should be overhauled or the rolling process should be adjusted. When measuring the clearance of the upper and lower work rolls or the clearance of the upper and lower work rolls and the upper and lower back-up rolls at the same time, the clearance between the bearing housing of each roll and the rolling mill can be comprehensively adjusted by referring to the above control strategy.

**Figure 14:**
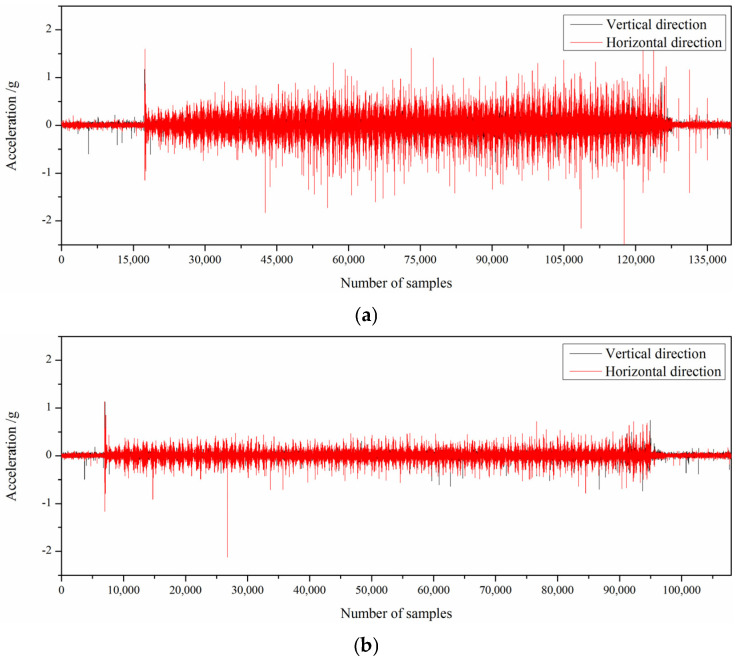
Vibration acceleration of the bearing housing on the operation side of the upper work roll. (**a**) Before clearance adjustment; (**b**) after clearance adjustment.

In the process of industrial experiment, the eddy current sensor was installed on the liner plate to measure the clearance between the bearing housing and the rolling mill stand, and the acceleration sensor was also installed on the work roll bearing housing to detect the vibration information of the rolling mill. The stability of rolling production is greatly improved by adjusting the clearance of F3 of 1580 mm hot tandem mill through the application of the control strategy of bearing housing clearance. The vibration of the rolling mill before and after adjustment is shown in Figure 14.

## 5. Conclusions

This paper proposed the concept of real-time measurement technology for the bearing housing clearance in rolling mills and designed real-time measurement methods for experimental rolling mills and industrial rolling mills, respectively. The laser ranging method is suitable for application in experimental rolling mills and is relatively simple to apply, but it is susceptible to various factors, such as the cooling water used in industrial fields. Preliminary data suggest that the measurement accuracy of the eddy current ranging method is not affected by cooling water. Moreover, because the sensors are mounted on the mill stand liners, this method is not affected by the frequent roll changes in the rolling mill. Therefore, this method is suitable for real-time clearance measurements in industrial rolling mills. The real-time measuring experiments were carried out in the laboratory and industrial field, respectively, and the results show that both measurement methods can obtain the clearance between the bearing housing and the rolling mill stand in real time. Furthermore, this paper develops a rolling mill bearing housing clearance control strategy. The control strategy can guide the fine management of rolling mill bearing housing clearance, which is of great significance to reduce mill vibration, reduce roll wear, and improve product quality.

## Data Availability

The experimental data can be obtained on request from xjk@ysu.edu.cn (J.X.). The experimental data were obtained through the National Engineering Research Center for Equipment and Technology of Cold Strip Rolling, Yanshan University. The experimental results are reproducible.
